# Impact of Intravenous Iron on Oxidative Stress and Mitochondrial Function in Experimental Chronic Kidney Disease

**DOI:** 10.3390/antiox8100498

**Published:** 2019-10-21

**Authors:** Faisal Nuhu, Anne-Marie Seymour, Sunil Bhandari

**Affiliations:** 1 School of Life Sciences (Biomedical), University of Hull, Kingston upon Hull HU67RX, UK; FISAFUN@YAHOO.CO.UK (F.N.); ANDI.SEYMOUR.19@GMAIL.COM (A.-M.S.); 2 Hull York Medical School & Department of Renal Medicine, Hull University Teaching Hospitals Trust, Anlaby Road, Kingston upon Hull HU32JZ, UK

**Keywords:** anaemia, chronic kidney disease, iron, mitochondrial dysfunction, oxidative stress

## Abstract

Background: Mitochondrial dysfunction is observed in chronic kidney disease (CKD). Iron deficiency anaemia (IDA), a common complication in CKD, is associated with poor clinical outcomes affecting mitochondrial function and exacerbating oxidative stress. Intravenous (iv) iron, that is used to treat anaemia, may lead to acute systemic oxidative stress. This study evaluated the impact of iv iron on mitochondrial function and oxidative stress. Methods: Uraemia was induced surgically in male Sprague-Dawley rats and studies were carried out 12 weeks later in two groups sham operated and uraemic (5/6 nephrectomy) rats not exposed to i.v. iron versus sham operated and uraemic rats with iv iron. Results: Induction of uraemia resulted in reduced iron availability (serum iron: 31.1 ± 1.8 versus 46.4 ± 1.4 µM), low total iron binding capacity (26.4 ± 0.7 versus 29.5 ± 0.8 µM), anaemia (haematocrit: 42.5 ± 3.0 versus 55.0 ± 3.0%), cardiac hypertrophy, reduced systemic glutathione peroxidase activity (1.12 ± 0.11 versus 1.48 ± 0.12 U/mL), tissue oxidative stress (oxidised glutathione: 0.50 ± 0.03 versus 0.36 ± 0.04 nmol/mg of tissue), renal mitochondrial dysfunction (proton/electron leak: 61.8 ± 8.0 versus 22.7 ± 5.77) and complex I respiration (134.6 ± 31.4 versus 267.6 ± 26.4 pmol/min/µg). Iron therapy had no effect on renal function and cardiac hypertrophy but improved anaemia and systemic glutathione peroxidase (GPx) activity. There was increased renal iron content and complex II and complex IV dysfunction. Conclusion: Iron therapy improved iron deficiency anaemia in CKD without significant impact on renal function or oxidant status.

## 1. Introduction

Iron-deficiency anaemia (IDA) is a major health problem worldwide. It is commonly associated with the progression of chronic kidney disease (CKD), affecting both quality of life and mortality [1,2,3,4]. Iron deficiency can exacerbate mitochondrial dysfunction and enhance oxidative stress in this patient population [5]. Mitochondrial proteins involved in oxidative phosphorylation (OXPHOS) pathway (respiratory complexes I-III) are iron complexes. Other mitochondrial enzymes, including aconitase of the Krebs cycle, require iron-sulphur (Fe-S) clusters (ISC) for their function [6,7]. Thus, derailment of mitochondrial iron homeostasis can result in human diseases associated with mitochondrial dysfunction such as Friedreich’s ataxia [8]. Iron deficiency distorts the tightly regulated biosynthesis of haem and ISC which are needed for mitochondrial function and aggravate mitochondrial oxidative stress [9]. Mitochondria are also increasingly recognised as major contributors to reactive oxygen species (ROS) production [10]. In dysfunctional mitochondria, the proton or potentially electron leak at complexes I and III causes excessive wasting of molecular oxygen into superoxide radical (O_2_•^−^). This can lead to the generation of more radicals (hydoxy (OH^−^) and peroxynitrite (ONOO^−^) radicals) [10]. Therefore, parenteral iron therapy employed in clinical practice as an integral component of managing anaemia of CKD may enhance mitochondrial function, and reduce overall oxidative stress (pro-oxidant versus anti-oxidant activity) without compromising renal function.

The mechanisms of progression of CKD are complex and multi-factorial and not completely elucidated. However, evidence implicating mitochondrial dysfunction in the initiation and progression of kidney disease comes from mitochondrial cytopathies [11]. Mitochondrial cytopathies (inherited or sporadic mtDNA mutations in mitochondrial genes) in kidneys lead to glomerular diseases, tubular defects and cystic kidney disease. Focal segmental glomerular sclerosis (FSGS), an example of glomerular disease emanating from mtDNA mutations, is a frequent cause of end stage kidney disease [12,13]. Other evidence of mitochondrial dysfunction in renal disease has come from in vivo and in vitro studies [14,15]. Mitochondrial dysfunction with reduced complex I, II and IV expression potentiated podocytes injury, impaired nephrin synthesis and increased ROS production [14]. Patients with iron deficiency anaemia from causes such as gastro-intestinal loss or menstruation do not appear to develop overt renal dysfunction. This suggests compensatory upregulation of mitochondrial function even in uraemic scenarios [16]. Although there is evidence of iron deficiency in CKD, tissue iron levels may vary between organs and may be preserved in the kidney minimising any detrimental renal effect.

Studies to date have provided evidence that the 5/6 nephrectomy used in this study is a suitable animal model of CKD showing enhanced vulnerability to oxidative stress and mitochondrial dysfunction [17]. However, limited information is available on the iron status of the model and response to therapy with intravenous (iv) iron. Most studies on the impact of iv iron are limited to acute systemic oxidative effects, disregarding their longer-term impact and benefits. Despite the central role of mitochondria (within the kidney) in the possible initiation and progression of CKD [18,19,20] and the integral role of iron in key mitochondrial proteins (aconitase, complex I, II or III), the impact of parenteral iv iron therapy as employed in clinical practice on mitochondrial function, oxidative stress and CKD progression has not been sufficiently characterised. 

The aim of this study was to determine the longer-term impact of iv iron administration on these parameters in an experimental animal model of CKD. The Ferumoxytol therapy (one form of available parenteral iron) protocol used in this study mimics that of clinical practice using an equivalent weight adjusted bolus injection at a dose of 510 mg in adults (i.e., 8–10 mg/Kg body weight) [21]. Ferumoxytol is a third-generation iron complex whose slow dissociation from the carbohydrate complex leads to the release of less “labile” iron, thus allowing a rapid bolus infusion of high doses clinically with favourable outcomes [22,23].

Initially, the aim was to ensure that the phenotype of the uraemic model exhibited IDA and further that iv iron therapy would ameliorate this deficiency. It was hypothesised that compromised renal mitochondrial function in this model would increase susceptibility to oxidative stress. Parenteral iron therapy could improve mitochondrial function of the remnant kidney, lessening the deleterious effect of oxidative stress. Therefore, iron status (systemic and tissue) and iron deficiency (ID) anaemia in the 5/6 nephrectomy model of CKD were studied and the impact of iv iron investigated. Hence, renal mitochondrial function in uraemia and renal and systemic oxidative stress before and following iron treatment were studied in detail.

## 2. Materials and Methods 

### 2.1. Induction of Experimental Uraemic Model 

All procedure and animals in this study were in accordance with the UK Animals (Scientific Procedure) Act 1986 and were approved by the University of Hull Ethical Review Process (No. PPL 70/7966). Experimental uraemia was induced in male Sprague-Dawley rats (obtained from Charles River Laboratories, Kent, UK) via a one-stage subtotal nephrectomy, as described previously [17].

Briefly, animals (250 g) were anaesthetised with 3% isofluorane in 3 L/min O_2_ and subsequently maintained on 2.5% isofluorane in 1 L/min oxygen. Depth of anaesthesia was confirmed using the pedal withdrawal reflex. Rimadyl was administered (4 mg/Kg body weight) pre-operatively via s/c injection for post-operative pain relief. A midline abdominal incision was made and the left kidney was exposed and decapsulated. Following clamping of the renal vasculature, approximately half of the kidney was excised comprised principally of cortical tissue. Haemorrhaging was controlled using Surgicel^®^ (Johnson & Johnson, Maidenhead, Berkshire, UK) before the remnant kidney was replaced. The right kidney was then exposed and decapsulated. Renal vasculature was ligated using a non-absorbable suture (Mersilk^®^ Johnson & Johnson, Maidenhead, Berkshire, UK) prior to excision of the kidney. Sterile isotonic saline (0.9% w/v) was administered into the abdominal cavity prior to closure to compensate for intraoperative fluid losses. The abdominal muscular layer was closed using an absorbable suture (Ethicon 3-0 Vicryl braided, Johnson & Johnson, Maidenhead, Berkshire, UK.) The dermal layer was closed with non-absorbable sutures (Ethicon 3-0 blue monofilament, Johnson & Johnson, Maidenhead, Berkshire, UK.) Sham animals were subjected to a sham procedure comprised of exposure and decapsulation of both kidneys. 

Iron therapy was initiated 6 weeks post-surgery by a single intravenous (iv) injection of ferumoxytol (supplied by Takaeda UK Ltd., Holborn, London, UK) at a dose of 10 mg/Kg body weight. All animals were maintained in individual cages for a total of 12 weeks post-surgery (six weeks after the iv iron) and pair fed (sham and uraemic) with a standard chow diet. Water was available ad libitum.

### 2.2. Model Characterisation

Urine samples collected over 24 h was filtered through Millex syringe-driven Filter unit (Merck KGaA, Darmstadt, Germany) and serum samples collected at week 12 were analysed using the RX Monza analyser (Randox, Antrim, UK) for creatinine, urea and total protein according to manufacturer’s protocol. Renal function was assessed by glomerular filtration rate (GFR) or creatinine clearance calculated using equation 1. Cardiac hypertrophy was evaluated by wet heart weight to tibia length ratio (HW/TL). Haematocrit was measured on an ABL77 Radiometer (Battery Universe Inc., USA) to confirm anaemia and packed cell volume (PCV) was subsequently measured to assess the impact of iv iron on anaemia. Briefly, heparinised blood samples were centrifuged and the ratio of packed red cell volume to whole blood volume calculated to give PCV. Iron status was determined from serum and urine biochemistry. Markers of iron status including serum iron, transferrin and total iron binding capacity were measured on the RX Monza analyser using Randox kits (Randox Laboratory Ltd., Crumlin, UK) as were urine samples. 

Serum ferritin was analysed using the Enzyme linked immunosorbent assay ELISA ferritin commercial Kit (Abcam, Cambridge UK). Hepatic, renal and cardiac tissue contents of non-bound and total iron were measured. Briefly, 200 mg of tissue or faecal excrete was extracted with 1 mL 7.35 mM sodium acetate trihydrate buffer (4.65 pH) for 10 min and centrifuged at 12,000× *g*, 4 °C in a microfuge (Scientific Laboratory Supplies, UK) for 10 min and the supernatant filtered through Millex syringe-driven Filter unit (Merck KGaA, Germany), the resultant filtrate and urine filtrate were analysed for non-bound iron on the RX Monza.

Hepatic, renal and cardiac iron contents were evaluated by total elemental iron analysis on Perkin Elmer Optima 5300DV emission ICP-OES instrument (PerkinElmer, Inc, Waltham, MA, USA). Briefly, Tissue or serum samples were extracted with concentrated HNO_3_ (Romil SpA trace metals, Cambridge UK) and digested in Teflon microwave vessels (MARS Xpress, CEM Ltd., Buckingham UK). The samples were allowed to cool, diluted with ultra-pure water and analysed on the Perkin Elmer Optima 5300 DV emission ICP instrument.

$$\begin{array}{c} \text{Creatinine Clearance}\\ \text{(mL/min/Kg body weight)} \end{array}=\frac{\left[\frac{\left[{\mathrm{Creatinine}}_{\mathrm{urine}}\right]}{\left[{\mathrm{Creatinine}}_{\mathrm{serum}}\right]}\times \frac{{\mathrm{Volume}}_{\mathrm{urine}}(\mathrm{mL})}{\mathrm{Time}\;(h)\times 60}\right]}{\text{Body Weight (kg)}}$$

### 2.3. Mitochondrial Function

Mitochondrial function was studied using the Seahorse XFp analyser (Agilent Technologies, Santa Clara, CA USA). The left kidney was excised, minced and mitochondria were isolated as previously described [17] in mitochondrial isolation buffer (containing 70 mM sucrose, 210 mM mannitol, 5 mM HEPES (4-(2-hydroxyethyl)-1-piperazineethanesulphonic acid), 1 mM EGTA (ethylene glycol-bis (betaaminoethylether)-N,N,N’N’-tetraacetic acid) and 0.5% (w/v) fatty acid-free BSA(bovine serum albumin), pH 7.2). The protein content was determined using the Bio-Rad protein assay. Mitochondrial coupling or electron flow experiments were carried out at 37 °C according to Roger et al. [24] and results were analysed on the Wave 2.3.0 software (Agilent Technologies, Santa Clara, CA USA). 

### 2.4. Oxidative Stress 

The impact of oxidative stress in this experimental model of CKD was evaluated by measuring lipid peroxidation through the levels of thiobarbuturic acid reactive substances (TBARS) according to modified method of Seljeskog et al. [25] and glutathione (both reduced and oxidised) using high performance liquid chromatography (HPLC) [26]. 

The kidney is a major source of glutathione peroxidase (GPx), an anti-oxidant; hence, its expression and activity is affected in CKD [4]. The activity of GPx was measured using the commercially available Ransel kit (Randox laboratories, Crumlin, UK) as per Paglia and Valentine [27]. The decrease in absorbance following the concomitant oxidation of NADPH (nicotinamide adenine dinucleotide phosphate hydrogen to NADP^+^ (nicotinamide adenine dinucleotide phosphate) was measured at 340 nm.

### 2.5. Transmission Electron Microscopy

The remnant kidney of uraemic animals was treated for ultrastructural analysis using a modified method of Rezzani et al. [28]. Briefly, renal tissue was fixed in 4% paraformaldehyde (pH 7.4) overnight at 4 °C and immersed in 2% osmium tetroxide at 4 °C for 1 hr. Tissue was dehydrated by immersion in graded ethanol and propylene oxide and embedded in Araldite-Epon resin. Representative blocks were taken subsequently with diamond knife and stained with uranyl acetate and lead citrate and observed using JEOL 2010 (JEOL, Inc, Peabody, MA, USA) high resolution transmission electron microscope at 80 kV to evaluate mitochondrial injury.

### 2.6. Statistical Analysis

The difference between two groups (uraemic and sham) was calculated using unpaired Student’s t test. Comparisons between treated and untreated groups were made using analysis of variance (ANOVA) on SPSS software. Data are presented as a mean ± standard error of the mean (SEM) or the standard deviations (SD). A p value less than 0.05 was considered statistically significant.

## 3. Results

### 3.1. Renal Function

**Figure 1:**
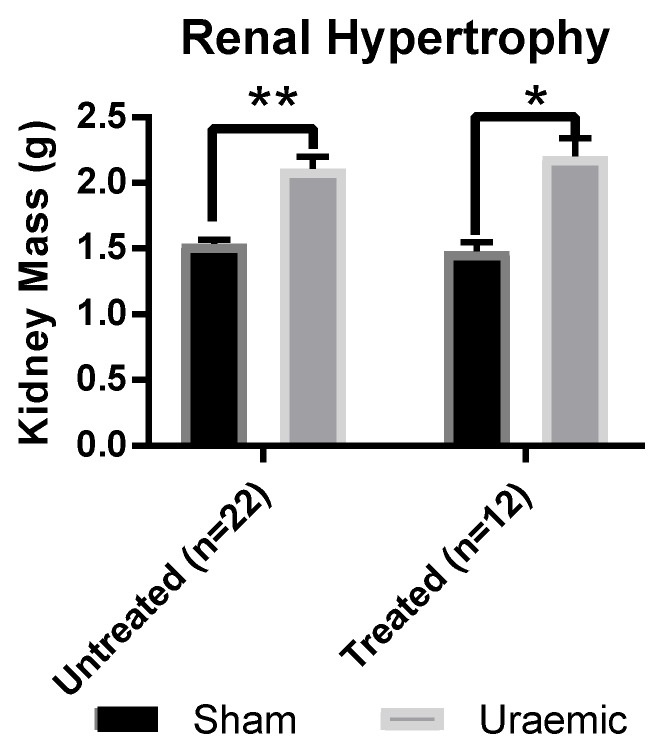
Renal hypertrophy in uraemic and sham animals (*n* = 22) and in animals exposed to intravenous (iv) iron therapy at six weeks was assessed by measuring left kidney mass. Data are presented as mean ± SEM, (* *p* < 0.05 and ** *p* < 0.01).

Uraemic animals demonstrated reduced renal clearance (estimated by glomerular filtration rate (GFR)) as evidenced by increased serum creatinine and urea concentrations (Table 1). Increased urinary and decreased serum protein (indicators of proteinuria) supported the renal insufficiency observed in this model. The remnant kidney underwent significant remodelling indicated by an increase of 37% and 48% kidney mass in untreated and iron treated uraemic groups, respectively (Figure 1). 

Uraemic animals exposed to iv iron therapy had a significantly lower serum creatinine (*p* < 0.05) and increased serum protein than those without iron. There was no change in the degree of proteinuria or renal dysfunction as a result of iron therapy in the uraemic group (Table 1).

### 3.2. Left Ventricular (LV) Hypertrophy

**Figure 2:**
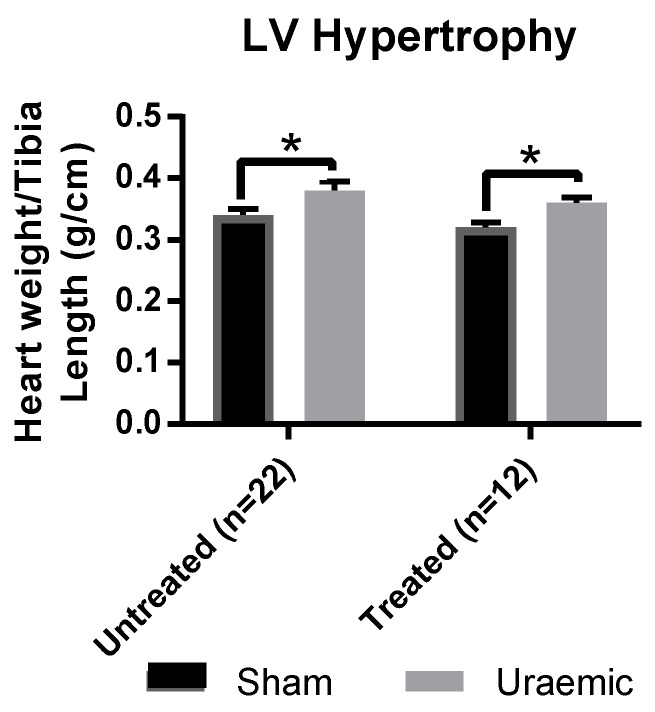
Cardiac hypertrophy 12 weeks post-surgical induction of uraemia was evaluated by measurement of heart weight to tibia length ratio in uraemia and sham animals with and without iv iron. Data are presented as mean ± SEM (* *p* < 0.05).

Induction of uraemia resulted in significant cardiac hypertrophy evidenced by an increased heart weight to tibia length ratio (HW/TL) (Figure 2). This is in agreement with previous observations [17]. Administration of iron did not impact on the extent of LV hypertrophy. 

### 3.3. Anaemia and Iron Status

**Figure 3:**
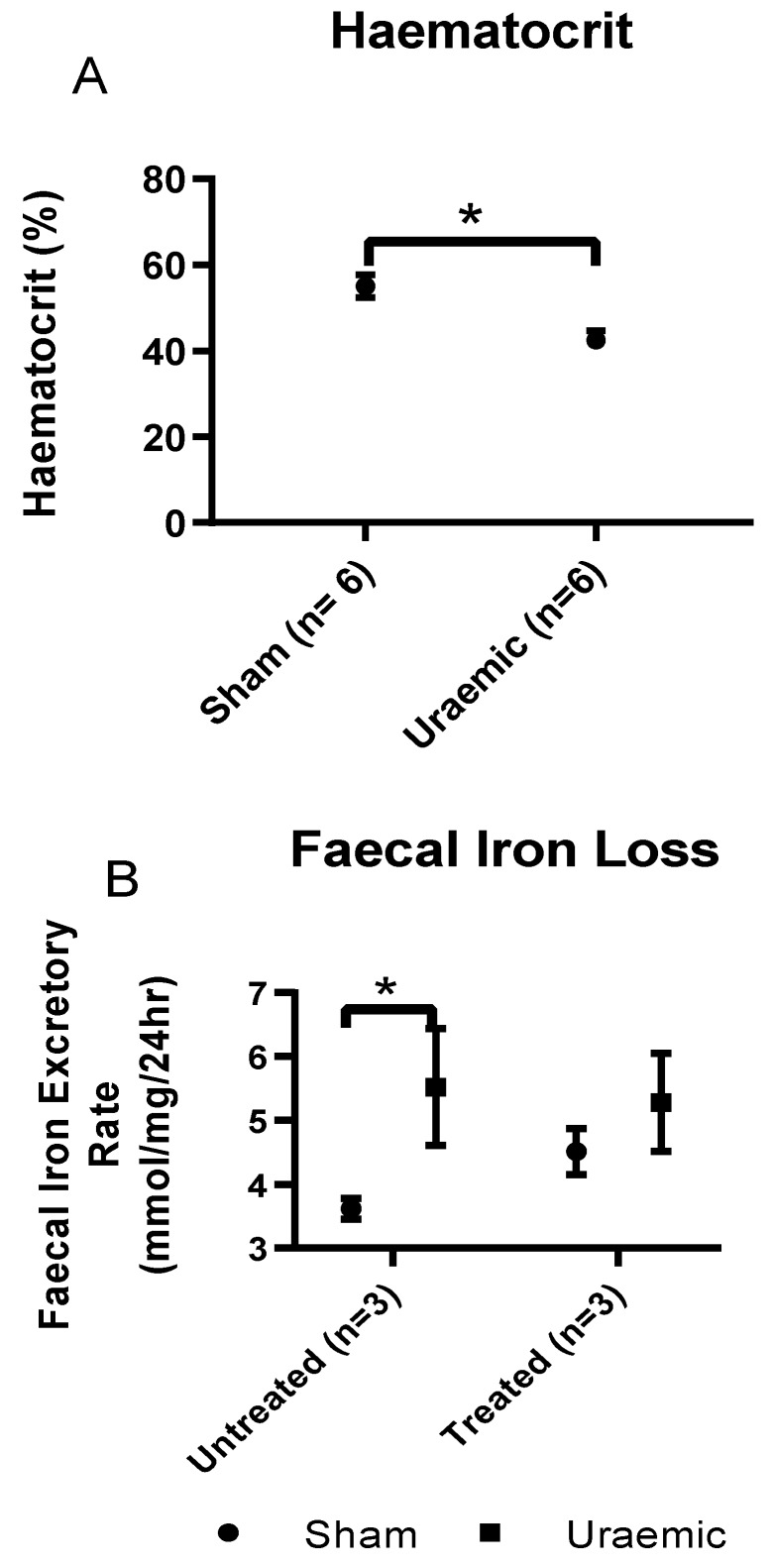

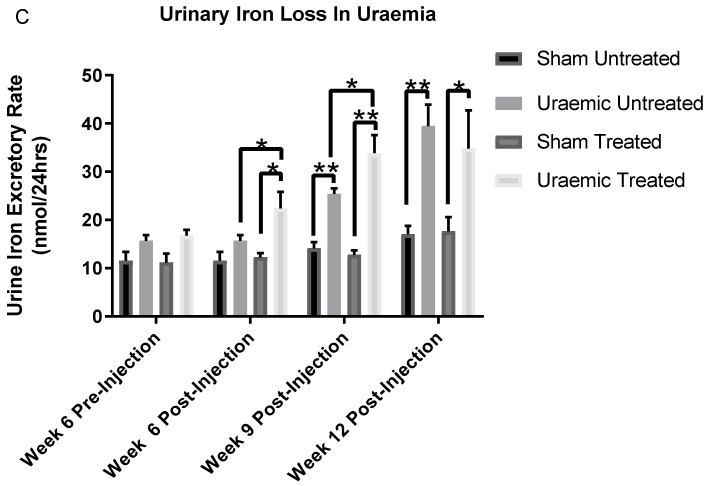
Iron analysis. (**A**) Haematocrit was measured to confirm anaemia; (**B**) Faecal Iron loss as a measure of iron malabsorption; (**C**) Urinary iron excretion was evaluated to study the cause of iron deficiency. Data are presented as mean ± SEM (* *p* < 0.05, ** *p* < 0.01).

**Figure 4:**
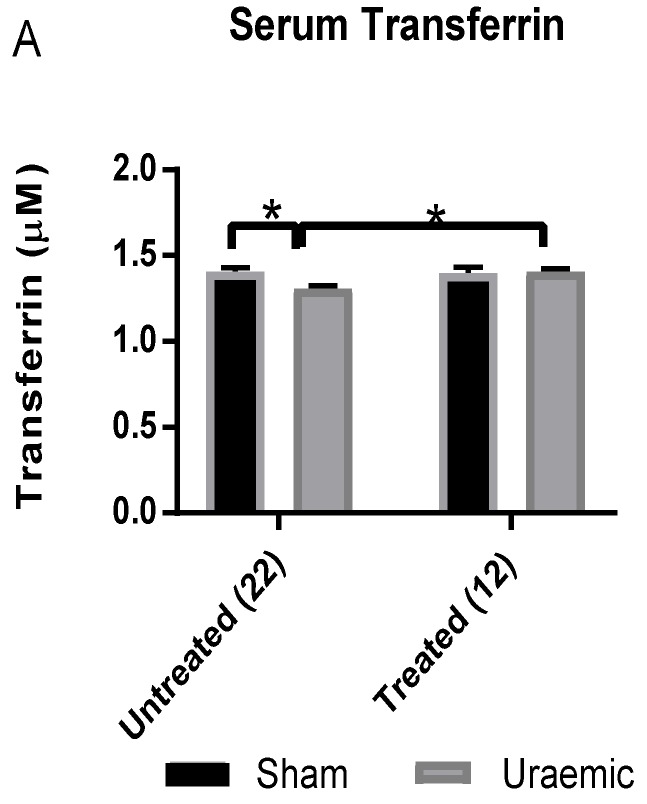

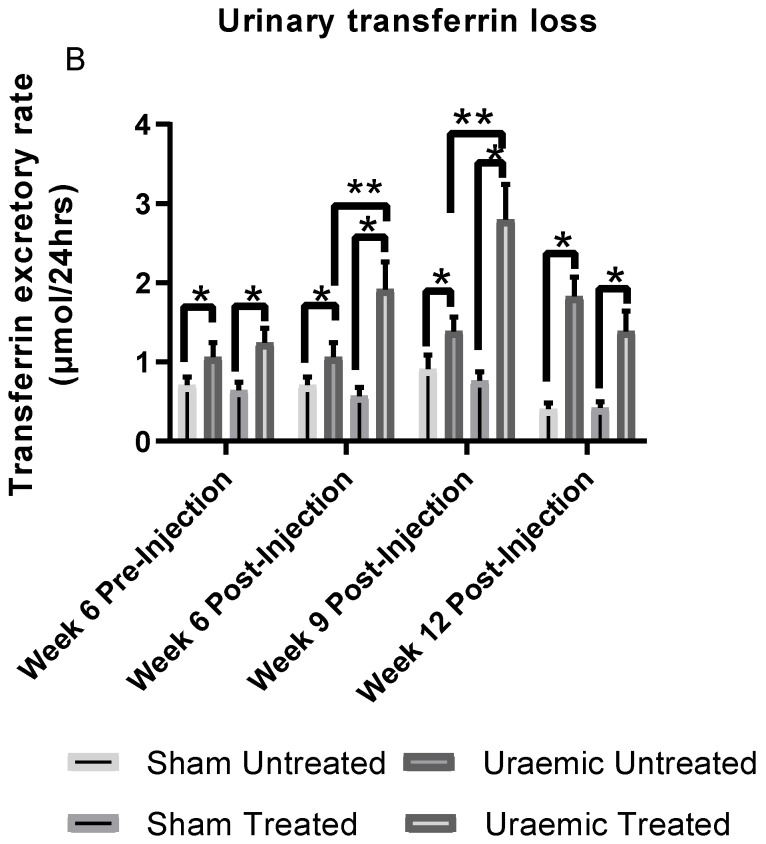
Transferrin analysis (**A**): Serum transferrin level and (**B**): Urinary transferrin loss at various stages of uraemia. Data are presented as mean ± SEM (* *p* < 0.05, ** *p* < 0.01).

The iron profile and packed cell volume in this model is given in Table 2. Uraemia was associated with anaemia characterised by a reduced haematocrit (Figure 3A) and a decreased serum iron. There was also increased faecal iron loss which may reflect reduced absorption or possible gastrointestinal bleeding and also increased urinary loss (Figure 3C). Serum transferrin was reduced alongside enhanced urinary loss (Figure 4) and lowered total iron binding capacity (TIBC). Liver iron stores and cardiac total iron concentrations were unchanged.

### 3.4. Impact of Iron Therapy

Intravenous iron therapy had a modest impact on iron deficiency anaemia in uraemic animals. There was restoration of serum transferrin and TIBC to a level similar to that observed in the sham group with an 8% increased PCV in the uraemic group without any significant change in the sham animals (Table 2). Increased faecal iron content was observed in the iron treated sham group (Figure 3B). Urinary transferrin and iron excretion in uraemic and sham operated groups were unchanged by week 12 in treated animals, in contrast to measurements 3 weeks after the iron bolus (Figure 4). Total iron measured by the elemental iron analysis in the uraemic remnant kidney was 46% higher in the treated group relative to the baseline data; this did not reach statistical significance. In cardiac tissue, there was a non-significant 22% reduction. Liver iron was increased significantly by 33% in the iron treated uraemic group relative to the iron treated sham group. This reflected a 45% increase relative to the untreated uraemic group (Table 2). 

### 3.5. Systemic and Renal Oxidative Stress

**Figure 5:**
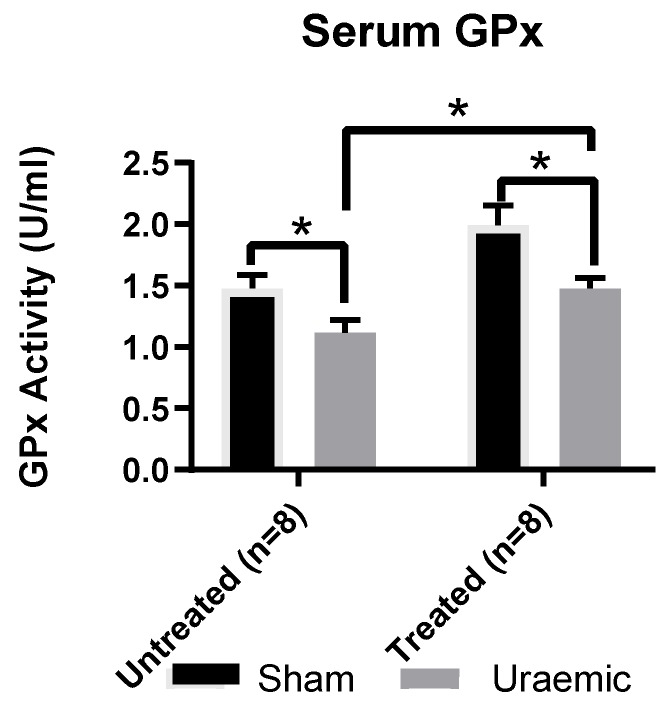
Serum glutathione peroxidase activity. Systemic anti-oxidant capacity was investigated through the measurement of glutathione peroxidase activity in the serum of uraemic and sham animals. Results are presented as mean ± SEM (* *p* < 0.05).

**Figure 6:**
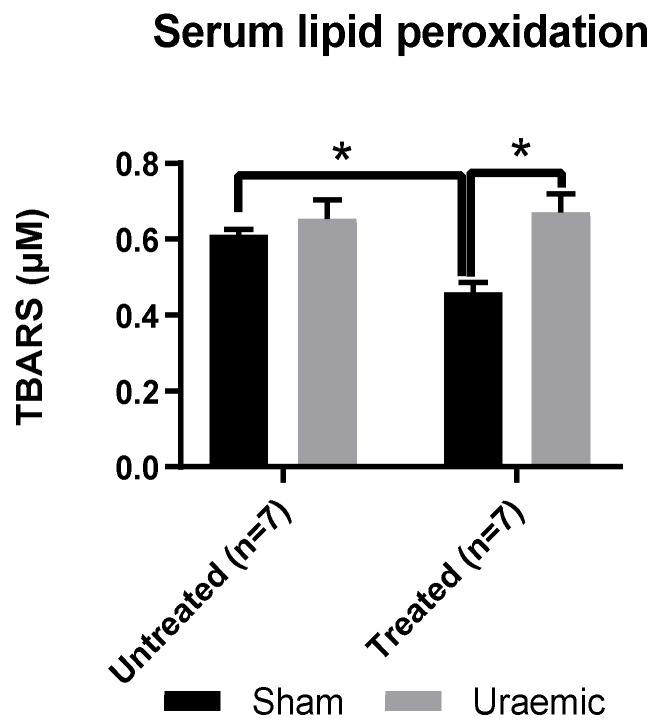
Serum TBARS. TBARS (thiobarbuturic acid reactive substances) were measured to access lipid peroxidation in uraemic and sham animals. Results are presented as mean ± SEM. (* *p* < 0.01).

**Figure 7:**
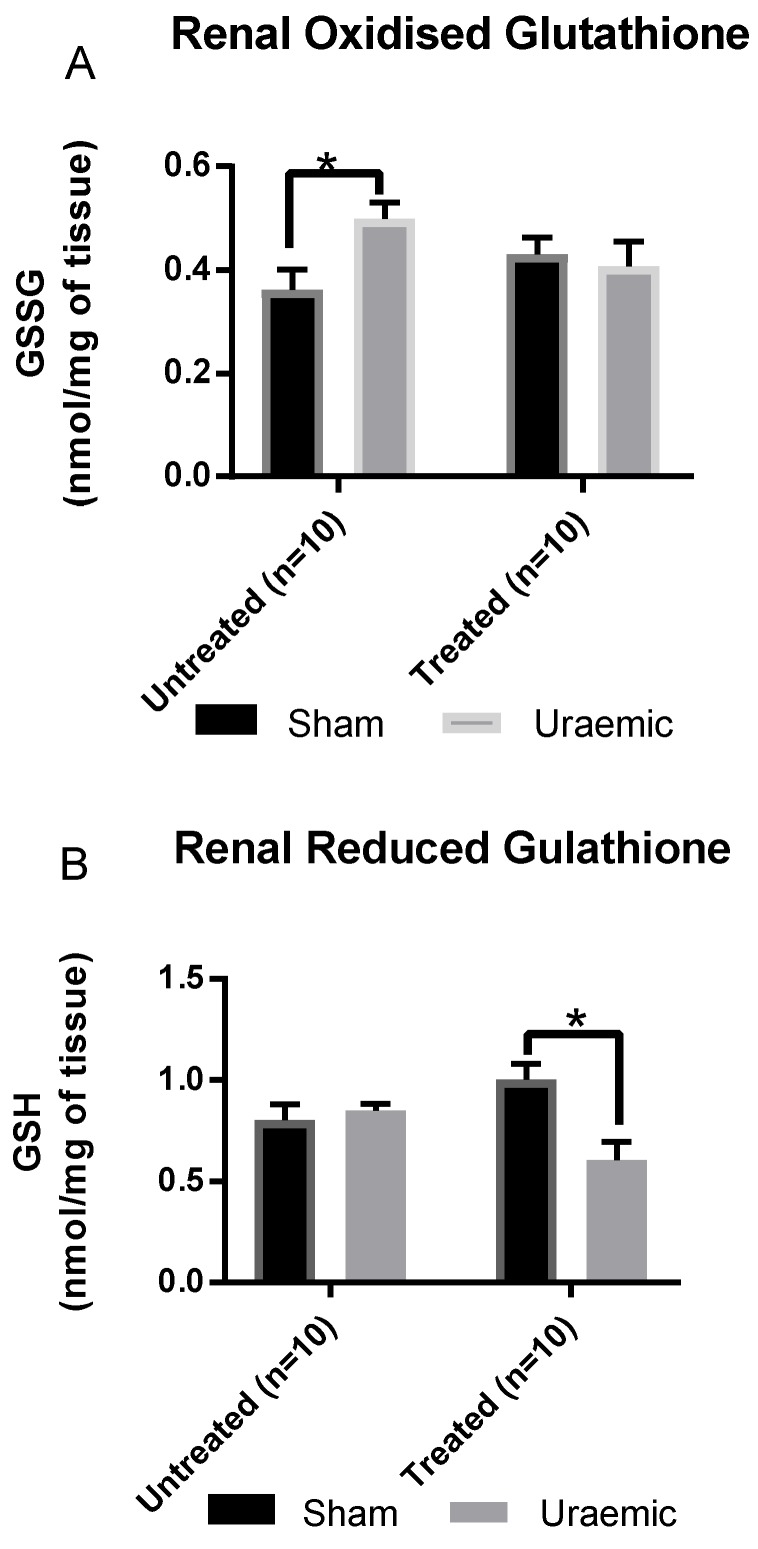
Endogenous antioxidant glutathione level in kidney tissue. (**A**) Renal oxidised glutathione in sham (*n* = 10) and uraemic animals (*n* = 10) with and without i.v. iron therapy; (**B**) renal reduced glutathione in sham (*n* = 10) and uraemic animals (*n* = 10) with and without i.v. iron therapy. Results are presented as mean ± SEM. (* *p* < 0.05).

This experimental model of CKD was associated with a 24% reduction of systemic GPx antioxidant activity (Figure 5) without evidence of systemic lipid peroxidation (Figure 6). This may reflect a possibly generalised reduction in protein synthesis or a marker of oxidative stress. There was an increased concentration of oxidised glutathione (GSSG) (Figure 7A) in the remnant kidney without any change in the reduced form (GSH) (Figure 7B). Treatment with iv iron was associated with reduced TBARS (*p* < 0.01) and upregulation of systemic GPx activity by 35% and 32% in sham and uraemic groups, respectively, relative to untreated but lower levels when comparing sham versus uraemic exposed to iv iron (Figure 5 and Figure 6). 

### 3.6. Renal Mitochondrial Function

**Figure 8:**
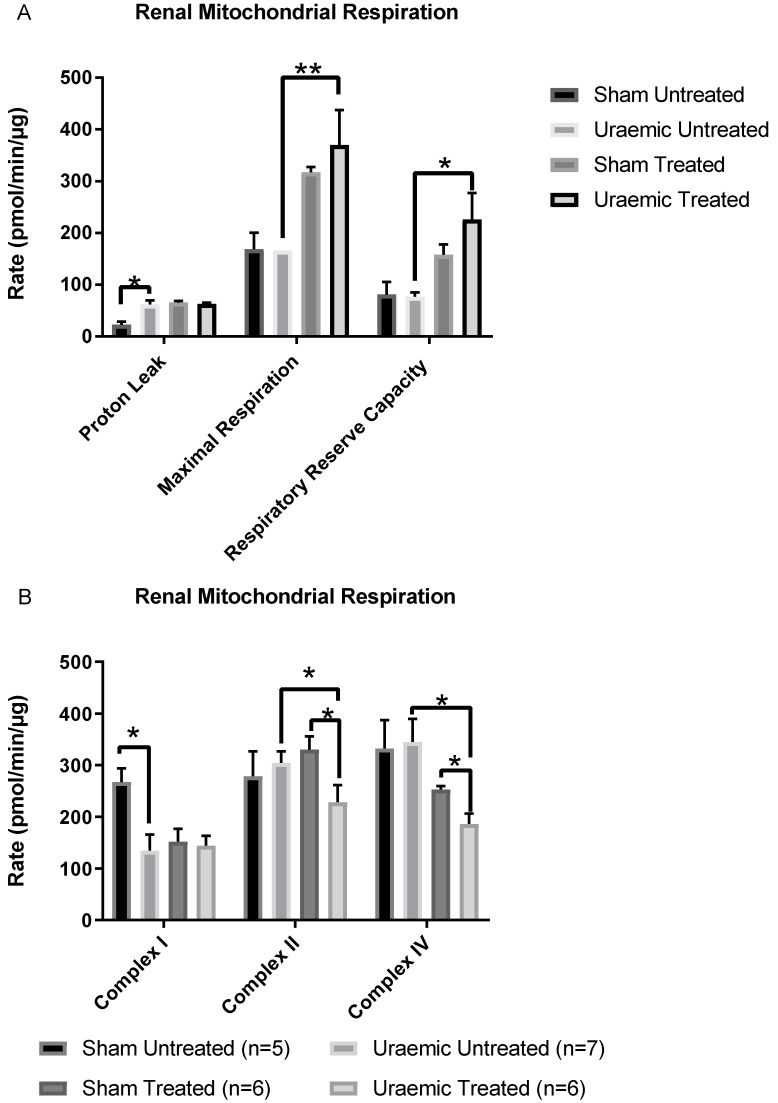
Isolated Mitochondrial measures of function including Respiratory rates and mitochondrial respiration in the presence of: (**A**) 0.5 µg mitochondrial protein, 10 mM succinate and 2 µM rotenone; (**B**) 0.6g mitochondrial protein, 10 mM pyruvate, 2 mM malate and 4 µM FCCP (carbonyl cyanide 4-(trifluoromethoxy) phenylhydrazone) with or without 10 mM malonate. In Fig 8B the electron transport chains complexes I, II and IV were measured. Results are presented as mean ± SEM. (* *p* < 0.05; ** *p* < 0.01). Proton leak = (minimum rate measured after Oligomycin injection) − (non-mitochondrial respiration rate or minimum rate measured after injection of Antimycin A); Maximal respiration = (maximal rate measured after FCCP injection) – (non-mitochondrial respiration rate); Respiratory reserve capacity = (maximal respiration – basal respiration).

Uraemic animals demonstrated that in renal tissue mitochondria, there was a significant increase in inefficiency (enhanced proton leak and complex I dysfunction) (Figure 8a). Iron therapy produced a mixed and complex result with no change in the protein leak but an increased maximal respiration and respiratory reserve capacity suggesting improved mitochondrial oxidative capacity. However, there was a reduction of complex II and complex IV driven respiration (Figure 8b), which would suggest a degree of mitochondrial dysfunction. 

## 4. Discussion

### 4.1. Induction of Uraemia and Impact of Iron Therapy

A significant deterioration of renal function was observed 12 weeks post-surgical induction of uraemia as indicated by the 124.6% and 92.7% increments in serum creatinine and urea, respectively. This is consistent with previous findings [29]. Progressive renal damage causes increased retention of creatinine and urea [30] resulting in decreased urinary levels as observed in Table 1. Impairment of kidney function as a result of persistent and progressive renal damage increases glomerular permeability and decreases tubular protein and fluid reabsorption. This in turn explains the 100% and 94% increments in urinary protein and volume, respectively. Weight gain 12 weeks post-surgery was similar between sham and uraemic groups, showing that malnutrition or loss of muscle mass reported in patients with CKD [31] was less likely to be a confounding factor. The elevated protein loss in urine correlated with a reduction in serum total protein. 

The remnant left kidney of uraemic animals underwent compensatory hypertrophy indicated by increased kidney weight without affecting renal function (measured by GFR, Table 1). This change in the remnant kidney could be an adaptive response in an attempt to “normalise” or improve renal function, albeit with limited success. Previous work from this group has demonstrated progressive compensatory hypertrophy of the remnant kidney at week 6 (18.8%; *p* < 0.05), with diminishing function [32]. 

Parenteral iron treatment did not affect renal function, but increased serum total protein and transferrin concentrations. There are several potential safety issues concerning iv iron therapy including increased oxidative stress, infection and proteinuria as biomarkers [33,34,35]. Nephrotoxicity of iv iron therapy is dependent on the iron formulation as reported by Agarwal et al. [36]. These authors reported that unlike ferric gluconate, iron sucrose produced a 78% increased proteinuric response that was unaltered following repeated doses of iv iron. This was consistent with other reports of worsening proteinuria in response to iron sucrose therapy but not ferric gluconate [37]. The lack of significant change in urinary proteins levels by 6 weeks post therapy indicated that ferumoxytol did not elicit a chronic proteinuria effect in uraemic animals in this study. This is not unsurprising given that there are known physiochemical differences between iron preparations; hence, this confounder cannot be excluded [38]. 

### 4.2. Anaemia in Uraemia and the Effect of Iron

Anaemia significantly increases the risk of morbidity and mortality in CKD [39]. Iron deficiency anaemia in CKD is associated with diminished cytochrome c oxidase activity, decreased mitochondrial oxidative capacity and reduced total anti-oxidant capacity resulting in enhanced mitochondrial oxidative stress [40]. Therefore, anaemia correction by erythropoietin and/or iron replenishing therapy is an integral component in the management of anaemia of CKD. The observation of reduced haematocrit as an indicator of anaemia is consistent with previous findings, which was improved following erythropoietin treatment [29]. Previous data indicated an inverse correlation between serum creatinine and haematocrit, suggesting the degree of anaemia is related to the severity of renal dysfunction [32]. Iron replenishment therapy via iv administration of iron complexes such as the third generation preparations (ferric carboxymaltose, iron isomaltoside and ferumoxytol) and the older formulations (low molecular weight iron dextran, iron sucrose and ferric gluconate) has proven to be effective in correcting iron deficiency anaemia in CKD [41,42].

Experimental uraemia resulted in a biomarker profile comparable to the clinical scenario of anaemia of inflammatory/chronic disease characterised by decreased serum iron with reduced serum TIBC [43]. This, together with maintenance of ferritin levels and liver iron content in sham and uraemic groups, indicates the inability to access stored iron in uraemia, similar to the classical setting of a pro-inflammatory state [44]. In absolute iron deficiency, reduction of serum iron would indicate a more readily available transferrin for iron binding (giving rise to increased TIBC). The reduction of TIBC found here can partly be explained by the decreased circulating transferrin (perhaps partly related to the reduction in protein concentrations). This observation was in agreement with the report of Alfrey and Hammond [45] where serum iron and transferrin decreased rapidly following the induction of nephrotoxic syndrome. The investigators also observed increased urinary iron and transferrin loss similar to this study’s observations. Urinary transferrin loss at week 12 of uraemia was 241% greater in uraemic animals compared to sham. Urinary iron loss increased by 66%. The present study has shown an inverse relationship between serum and faecal iron. No sign of blood in the faecal excreta of uraemic animals is indicative of little or no intestinal bleeding. Hence, the increase in faecal iron excretion is suggestive of malabsorption of iron or impaired absorption in the gut. The evidence also highlights increased urinary iron loss in relation to the severity of renal dysfunction. These two factors may be critical in determining the mechanism of iron deficiency anaemia in this model of CKD. 

Studies have shown the elevation of inflammatory markers and concomitant upregulated expression of hepcidin [46,47] can lead to functional iron deficiency (ID) anaemia in CKD [48,49]. Given the central role of hepcidin in iron metabolism, assessment of serum, urinary and hepatic hepcidin levels and inflammatory markers such as interleukins (IL-1 and IL-6) could provide insight into the aetiology of iron deficiency. Nonetheless, uraemia may mediate hepcidin over-secretion resulting in enhanced destruction of ferroportin [50] and accumulation of dietary absorbed iron in the enterocytes. Subsequent loss of the iron via enterocyte shedding could explain the 52.7% increment in faecal iron in uraemic animals and the ensuing ID. However, hepcidin over-secretion may also prevent the release of iron from hepatocyte stores, causing an increase in hepatic iron, a phenomenon not observed in the present study. ID impairs red blood cells (RBCs) production, which could explain the presence of anaemia in this study. Persistent anaemia leads to significant compensatory left ventricular hypertrophy (as observed in this model of CKD in Figure 2), which eventually results in congestive heart failure in later stages of uraemia [51]. 

Iron therapy in the present study resulted in a significantly increased PCV (8%) in the uraemic group. Two prospective randomised studies found that a greater increase in haemoglobin above baseline was observed in CKD patients on ferumoxytol therapy than those on oral iron [52,53]. Parenteral ferumoxutol therapy in a randomised study was effective in raising the mean haemoglobin level and tolerable in patients in whom oral iron was ineffective [53]. Iron treatment restored serum transferrin levels in uraemic animals without changing urinary transferrin loss, suggesting increased synthesis. Serum iron remained low in the iron treated group with significant reduction in the sham group which could in part be due to the enhanced urinary loss. Iron therapy results in a rapid increase in circulating iron levels, which might trigger a homeostatic response [54]. This includes increased expression of transferrin and the transferrin receptor, which mediates cellular iron uptake. It is expressed at low levels in hepatocytes and is down-regulated in response to iron. Liver overload occurs when transferrin is completely saturated and hepatocytes internalise non-transferrin-bound iron present in the bloodstream explaining the observation of increased liver iron. Consequently, over time, total iron binding capacity increases as serum iron falls, as observed here.

Despite maintenance in total cardiac iron content, iron therapy led to a trend in reduced non-bound iron in cardiac and hepatic tissues that accounts for the cytotoxicity of iv iron. Intravenous iron therapy was associated with increased accumulation of iron in the liver. This is similar to the clinical evidence where iv iron therapy in haemodialysis patients was associated with iron accumulation in the spleen and liver but not the heart [55]. The accumulated iron in the spleen and liver serves as reserves that maintained erythropoiesis and could explain the amelioration of anaemia in the present study 6 weeks after bolus iv iron injection. The urinary iron loss alongside the transferrin loss observed in uraemic animals was exacerbated following therapy. There were no mortalities or observable side effects associated with ferumoxytol therapy, though adverse effects such as dizziness, pruritus, headache, fatigue and nausea have been reported in clinical studies [56]. These could not be reliably assessed in this rat model of CKD.

### 4.3. Oxidative Stress

This model of CKD was accompanied by a reduction of serum GPx activity suggesting depression in systemic antioxidant capacity. This did not translate into systemic oxidative stress, and thus, may be an indication of an early stage of the disease process or a degree of adaptation of the oxidative system to uraemia. The observation of reduced GPx was in line with the reports of Romeu et al. [57]. Serum TBARS in uraemic group were comparable to sham, indicating no systemic oxidative damage at this stage of CKD. This does not exclude an initial acute effect as a result of uraemia but may also suggest compensation or adaptation during the first weeks. This is in contrast to reports of increased systemic TBARS and GSSG in patients with CKD [58]. Patients with CKD were characterised by increased markers of systemic oxidative stress [59], such as increased level of serum lipid peroxidation markers [58], GSSG [57] and protein carbonyls [60]. Blood is rich in both enzymatic and non-enzymatic antioxidant components to detoxify reactive oxygen species (ROS). However, in the event of increased cellular oxidative activity, excess ROS can spill into the circulation initiating a cycle of ROS induced ROS generation; ROS mediated loss of anti-oxidant capacity and culminate in overt systemic oxidative stress [61]. Therefore, the depression in systemic GPx activity here could be an early event that increases the vulnerably to systemic oxidative stress later in CKD if there is no adequate compensation. Increased kidney GSSG levels were found in uraemic animals in this study. GSH is a reductant that reduces free radicals in oxidation reactions leading to the generation of GSSG. Hence, increased GSSG levels may be indicative of enhanced pro-oxidant activity. 

The improved iron deficiency anaemia by iv ferumoxytol in the present study enhanced GPx activity considering the increased evidence of systemic lipid peroxidation, indicating potential long-term benefit of iv iron to CKD patients with ID anaemia. There are conflicting reports regarding the impact of iv iron on oxidative stress in CKD patients [5], as it is associated with some increase or no effect on systemic oxidative stress [62,63]. These conflicting reports could be attributed to differences in iron formulation and the dosage used [64,65]; severity of the disease and whether or not patient is on dialysis [66]. Following iv administration of 100 mg iron sucrose to stage 3 or 4 CKD patients, Agarwal et al. [66] found increased lipid peroxidation within 15 to 30 min that completely resolved within 24 h. Unequivocally, iv iron precipitates acute oxidative toxicity as demonstrated by the recent report of Kuo et al. [67]. In their in vitro studies, the investigators reported transient increases in ROS generation between 1–3 h and enhanced NADPH oxidase activity within 30–60 min following 160 μg/mL iron sucrose supplementation, which normalised after 4 h. Administration of the antioxidant, n-acetylcysteine, together with iron therapy attenuated ROS production and its associated endothelial dysfunction [67]. Hence, antioxidant therapy use concomitantly with iv iron clinically may alleviate acute iron oxidative toxicity and facilitate the appreciation of the many chronic benefits highlighted in this study.

### 4.4. Mitochondrial Dysfunction

**Figure 9:**
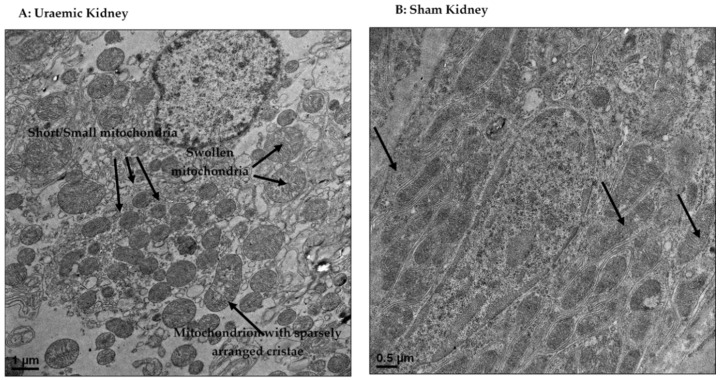
Transmission electron microscopic representation of uraemic mitochondria (**A**) with evidence of mitochondrial fragmentation (fission) as indicated by the presence of smaller mitochondrial spheres; swollen mitochondria with sparsely arranged cristae relative to sham mitochondria (**B**). Arrows indicate mitochondria.

The remnant kidney of uraemic animals demonstrated mitochondrial dysfunction as illustrated by increased proton/electron leak. Proton/electron leaks whether due to specific uncoupling proteins or non-specific transfer of protons/electrons into the matrix results in loss of membrane potential [68,69]. The conversion of ADP to ATP by mitochondrial complex V is dependent on membrane potential, the loss of which severely compromises bioenergetics and could partly explain the exacerbation of kidney dysfunction in this study. The kidney is a metabolic organ requiring high energy (ATP) to drive its function including active reabsorption of solutes [70]. Tubular cells are rich in mitochondria, and consequently, any injury would compromise renal function and precipitate CKD [71]. The uraemic kidney in this study demonstrated several structural and biogenetic abnormalities. Transmission electron microscopic study of the remnant kidney (uraemic) revealed existence of swollen mitochondria with loss of cristae and matrix density (Figure 9). Mitochondrial matrix swelling and loss of cristae membranes were evident after renal ischaemia in rats, affecting ATP regeneration after reperfusion [72]. 

Our observation of mitochondria with rounded morphology in the uraemic kidney is similar to the report of Lan et al. [73]. In a similar study, the remnant kidney following 5/6 nephrectomy showed fragmented and dysmorphic mitochondria with evidence of swelling and disrupted cristae architectures [20]. The authors reported diminished mitochondrial function and reduced antioxidant capacity alongside oxidative stress similar to the observation presented here. Under normal conditions, antioxidant enzymes (such as glutathione peroxidase, peroxyredoxin, glutaredoxin 2, thioredoxin and catalase) protect the mitochondria from ROS attack, and hence prevent membrane damage and peroxidation [74]. However, in conditions of diminished antioxidant activity as observed in this model of CKD, damage to mitochondrial inner membrane can occur, leading to the inexorable decline in mitochondrial function and worsen kidney function and its associated complications. 

The mechanisms underlying loss of cristae in the present model are unclear but ongoing work is focused on changes in mitochondrial membrane cardiolipin (CL) content and remodelling as possible factors. Indeed, there is increased vulnerability of CL to peroxidation owing to its close proximity to site of ROS production and in part due to its relative high content of unsaturated fatty acyl chains [75,76]. This could explain the increased evidence of mitochondrial fission as shown by the presence of fragmented mitochondria (Figure 9a) in the uraemic kidney. Mitochondrial fission involved the division of the mitochondrion into two daughter organelles, and when in excess, results in mitochondrial fragmentation. Mitochondrial fragmentation is increasingly evident in many kidney diseases [77,78]. 

Uraemic animals showed mitochondrial complex I respiratory dysfunction in the remnant kidney, which was not improved by iron therapy. In addition, iron therapy was associated with reduced complex II and complex IV driven respiration that could be related to the increased accumulation of iron observed in the remnant kidney of uraemic animals. Indeed, dysfunctional mitochondria can potentiate excessive generation of ROS. As reported by Zhu et al. [79], mitochondrial dysfunction underlined by decreased expression of complexes I, II and IV was associated with increased generation of ROS and oxygen consumption which is not coupled to ATP production. However, despite these cellular changes iv iron appeared to increase maximal respiration and respiratory reserve capacity suggestion a degree of mitochondrial adaptation or upregulation of numbers or function at this stage of CKD. This requires further work to elicit the complex changes occurring within the kidney.

## 5. Conclusions

The findings presented here have confirmed the phenotype of iron deficiency anaemia in experimental uraemia. It has also been demonstrated that there is both mitochondrial functional adaptation and cellular dysfunction and evidence of changes in renal and systemic oxidative stress. These findings suggest that mitochondrial activity is increased in the remnant kidney tissue to compensate for reduced renal mass. This is supported by the modest decrease in renal function despite the removal of a large majority of renal tissue. The data also suggest that kidney injury may impact on mitochondrial biogenesis, ultra-structure organisation and function. Further work assessing mitochondrial mass will help clarify this further. Correction of iron deficiency anaemia with a bolus of ferumoxytol in uraemic animals was associated with a partial restoration of systemic antioxidant GPx activity and importantly no deterioration in renal function or increase in proteinuria. The data suggest that timely administration of iv iron may help to alleviate the complications of iron deficiency relating to oxidative stress in CKD patients. This study further highlights the need for therapies targeting mitochondria specifically as a part of routine CKD management, in addition to the iron and antioxidant therapies. It is not known if similar observations occur with other iron preparations which may have subtle different physiochemical properties.

## Figures and Tables

**Table 1:** Markers of renal function in sham and uraemic animals with and without iv iron. Data are presented as mean ± SEM. GFR (glomerular filtration rate) * *p* < 0.05; sham versus uraemic; ǂ *p* < 0.05; uraemic untreated versus treated.

Characterisation of Uraemic model in CKD at week 12				
	Untreated (*n* = 22)		Iron Treated (*n* = 12)	
	Sham	Uraemic	Sham	Uraemic
Weight gain over 12 weeks (g)	301.5 ± 11.5	301.0 ± 13.7	297.4 ± 14.6	303.3 ± 12.8
Serum				
Creatinine (µM)	38.43 ± 1.73	86.32 ± 4.09 *	50.32 ± 1.65	76.11 ± 2.86 *ǂ
Urea (mM)	10.81 ± 0.94	20.83 ± 1.60 *	8.70 ± 0.31	20.68 ± 1.79 *
Total protein (g/dL)	5.60 ± 0.14	5.13 ± 0.14 *	6.67 ± 0.22	6.67 ± 0.08 ǂ
24 h Urinary	(*n* = 9)	(*n* = 9)	(*n* = 5)	(*n* = 5)
Total Protein (g/L)	1.44 ± 0.17	2.89 ± 0.31 *	1.39 ± 0.11	2.97 ± 0.30 *
Creatinine (mM)	17.02 ± 1.27	11.61 ± 1.08 *	18.41 ± 3.74	9.21 ± 1.01 *ǂ
Total volume (mL)	14.33 ± 1.75	23.75 ± 2.68 *	16.40 ± 2.16	21.6 ± 2.86 *
GFR (ml/min/Kg body weight)	7.86 ± 0.84	3.96 ± 1.03 *	7.53 ± 0.97	3.22 ± 0.76 *

**Table 2:** Markers of iron status in sham and uraemic animals with and without iron therapy. Data are presented as mean ± SEM. * *p* < 0.05; sham versus uraemic. ǂ *p* < 0.05; uraemic untreated versus treated. TIBC = total iron bonding capacity.

Anaemia Characterisation at week 12				
	Untreated (*n* = 22)		Iron Treated (*n* = 12)	
	Sham	Uraemic	Sham	Uraemic
Serum				
TIBC (µM)	29.46 ± 0.83	26.43 ± 0.72 *	33.55 ± 1.16	29.09 ± 0.79 *ǂ
Ferritin _(*n* = 8)_ (µM)	0.12 ± 0.03	0.13 ± 0.03	0.12 ± 0.01	0.11 ± 0.03
Iron _(*n* = 11)_ (µM)	46.38 ± 1.44	31.11 ± 1.80 *	32.82 ± 0.97	28.89 ± 1.65
Packed cell volume	0.58 ± 0.02	0.50 ± 0.01 *	0.57 ± 0.01	0.54 ± 0.03
Tissue Iron (*n* = 7) (micromole/g of tissue)				
Liver stores	2.91 ± 0.22	3.26 ± 0.18	3.57 ± 0.27	4.72 ± 0.20
Liver (non-bound)	0.34 ± 0.03	0.38 ± 0.02	0.34 ± 0.02	0.31 ± 0.00*ǂ
Kidney	2.00 ± 0.27	1.90 ± 0.10	1.90 ± 0.08	2.77 ± 0.40
Heart (×10^−3^) (total)	1.57 ± 0.07	1.85 ± 0.18	1.40 ± 0.07	1.55 ± 0.07
Heart (×10^−3^) (non-bound)	0.30 ± 0.02	0.33 ± 0.02	0.26 ± 0.03	0.26 ± 0.02 ǂ

## References

[1] Mehdi U., Toto R.D. (2009). Anemia, Diabetes, and Chronic Kidney Disease. Diabetes Care.

[2] Kovesdy C., Trivedi B., Kalantar-Zadeh K., Anderson J. (2006). Association of anemia with outcomes in men with moderate and severe chronic kidney disease. Kidney Int..

[3] Regidor D.L., Kopple J.D., Kovesdy C.P., Kilpatrick R.D., McAllister C.J., Aronovitz J., Greenland S., Kalantar-Zadeh K. (2006). Associations between Changes in Hemoglobin and Administered Erythropoiesis-Stimulating Agent and Survival in Hemodialysis Patients. J. Am. Soc. Nephrol..

[4] Bhandari S. (2011). Beyond efficacy and safety-the need for convenient and cost-effective iron therapy in health care. NDT Plus.

[5] Nuhu F., Bhandari S. (2018). Oxidative Stress and Cardiovascular Complications in Chronic Kidney Disease, the Impact of Anaemia. Pharmaceuticals.

[6] Horowitz M.P., Greenamyre J.T. (2010). Mitochondrial Iron Metabolism and Its Role in Neurodegeneration. J. Alzheimer’s Dis..

[7] Urrutia P.J., Mena N.P., Núñez M.T. (2014). The interplay between iron accumulation, mitochondrial dysfunction, and inflammation during the execution step of neurodegenerative disorders. Front. Pharmacol..

[8] Abeti R., Parkinson M.H., Hargreaves I.P., Angelova P.R., Sandi C., Pook M.A., Giunti P., Abramov A.Y. (2016). Mitochondrial energy imbalance and lipid peroxidation cause cell death in Friedreich’s ataxia’. Cell Death Dis..

[9] Vaubel R.A., Isaya G. (2013). Iron-Sulfur Cluster Synthesis, Iron Homeostasis and Oxidative Stress in Friedreich Ataxia. Mol Cell Neurosci.

[10] Wong H.-S., Dighe P.A., Mezera V., Monternier P.-A., Brand M.D. (2017). Production of superoxide and hydrogen peroxide from specific mitochondrial sites under different bioenergetics conditions. J. Biol. Chem..

[11] Hall A., Unwin R., Hanna M., Duchen M., Duchen M. (2008). Renal function and mitochondrial cytopathy (MC): More questions than answers?. QJM: Int. J. Med..

[12] Kiffel J., Rahimzada Y., Trachtman H. (2011). Focal segmental glomerulosclerosis and chronic kidney disease in pediatric patients. Adv. Chronic. Kidney Dis..

[13] Liu J., Xie J., Zhang X., Tong J., Hao X., Ren H., Wang W., Chen N. (2017). Serum C3 and Renal Outcome in Patients with Primary Focal Segmental Glomerulosclerosis. Sci. Rep..

[14] Zhu C., Huang S., Yuan Y., Ding G., Chen R., Liu B., Yang T., Zhang A. (2011). Mitochondrial dysfunction mediates aldosterone-induced podocyte damage: A therapeutic target of PPARγ. Am. J. Pathol..

[15] Granata S., Gassa A.D., Tomei P., Lupo A., Zaza G. (2015). Mitochondria: A new therapeutic target in chronic kidney disease. Nutr. Metab..

[16] Cummings B.S., Parker J.C., Lash L.H. (2000). Role of cytochrome P450 and glutathione S-transferase alpha in metabolism and cytotixicty of trichlorethylene in rat kidney. Biochem. Pharmacol..

[17] Taylor D., Bhandari S., Seymour A.-M.L. (2015). Mitochondrial dysfunction in uremic cardiomyopathy. Am. J. Physiol. Physiol..

[18] Szeto H.H. (2017). Pharmacologic Approaches to Improve Mitochondrial Function in AKI and CKD. J. Am. Soc. Nephrol..

[19] Bigelman E., Cohen L., Aharon-Hananel G., Levy R., Rozenbaum Z., Saada A., Keren G., Entin-Meer M. (2018). Pathological presentation of cardiac mitochondria in a rat model for chronic kidney disease. PLoS ONE.

[20] Chen J.-F., Liu H., Ni H.-F., Lv L.-L., Zhang M.-H., Zhang A.-H., Tang R.-N., Chen P.-S., Liu B.-C. (2013). Improved Mitochondrial Function Underlies the Protective Effect of Pirfenidone against Tubulointerstitial Fibrosis in 5/6 Nephrectomized Rats. PLoS ONE.

[21] Hetzel D., Strauss W., Bernard K., Li Z., Urboniene A., Allen L.F. (2014). A Phase III, randomized, open-label trial of ferumoxytol compared with iron sucrose for the treatment of iron deficiency anemia in patients with a history of unsatisfactory oral iron therapy. Am. J. Hematol..

[22] MacDougall I.C., Strauss W.E., McLaughlin J., Li Z., Dellanna F., Hertel J. (2014). A randomized comparison of ferumoxytol and iron sucrose for treating iron deficiency anemia in patients with CKD. Clin. J. Am. Soc. Nephrol..

[23] Schiller B., Bhat P., Sharma A. (2014). Safety and Effectiveness of Ferumoxytol in Hemodialysis Patients at 3 Dialysis Chains in the United States Over a 12-Month Period. Clin. Ther..

[24] Rogers G.W., Brand M.D., Petrosyan S., Ashok D., Elorza A.A., Ferrick D.A., Murphy A.N. (2011). High Throughput Microplate Respiratory Measurements Using Minimal Quantities Of Isolated Mitochondria. PLoS ONE.

[25] Seljeskog E., Hervig T., Mansoor M.A. (2006). A novel HPLC method for the measurement of thiobarbituric acid reactive substances (TBARS). A comparison with a commercially available kit. Clin. Biochem..

[26] Kand’Ár R., Žáková P., Lotková H., Kučera O., Červinková Z., Královcová P. (2007). Determination of reduced and oxidized glutathione in biological samples using liquid chromatography with fluorimetric detection. J. Pharm. Biomed. Anal..

[27] Paglia D.E., Valentine W.N. (1967). Studies on the quantitative and qualitative characterization of erythrocyte glutathione peroxidase. J. Lab. Clin. Med..

[28] Stacchiotti A., Favero G., Giugno L., Lavazza A., Reiter R.J., Rodella L.F., Rezzani R. (2014). Mitochondrial and Metabolic Dysfunction in Renal Convoluted Tubules of Obese Mice: Protective Role of Melatonin. PLoS ONE.

[29] Smith K., Semple D., Aksentijević D., Bhandari S., Seymour A.M. (2010). Functional and metabolic adaptation in uraemic cardiomyopathy. Front. Biosci..

[30] Smith K., Semple D., Bhandari S., Seymour A.M. (2009). The cellular basis of uraemic cardiomyopathy—A role of erythropoietin?. Eur. J. Heart Fail..

[31] Wang X.H., Mitch W.E. (2014). Mechanisms of muscle wasting in chronic kidney disease. Nat. Rev. Nephrol..

[32] Reddy V., Bhandari S., Seymour A.M. (2007). Myocardial function, energy provision, and carnitine deficiency in experimental uremia. J. Am. Soc. Nephrol..

[33] Auerbach M., Macdougall I.C. (2014). Safety of intravenous iron formulations: Facts and folklore. Blood Transfus.

[34] Agarwal R., Leehey D.J., Olsen S.M., Dahl N.V. (2011). Proteinuria Induced by Parenteral Iron in Chronic Kidney Disease—A Comparative Randomized Controlled Trial. Clin. J. Am. Soc. Nephrol..

[35] Del Vecchio L., Longhi S., Locatelli F. (2016). Safety concerns about intravenous iron therapy in patients with chronic kidney disease. Clin. Kid.

[36] Agarwal R., Rizkala A., Kaskas M., Minasian R., Trout J. (2007). Iron sucrose causes greater proteinuria than ferric gluconate in non-dialysis chronic kidney disease. Kidney Int..

[37] Besarab A., Levin A. (2000). Defining a renal anemia management period. Am. J. Kidney Dis..

[38] Bhandari S., Pereira D.I.A., Chappell H.F., Drakesmith H. (2018). Intravenous Irons: From Basic Science to Clinical Practice. Pharmaceuticals.

[39] Besarab A., Coyne D.W. (2010). Iron supplementation to treat anemia in patients with chronic kidney disease. Nat. Rev. Nephrol..

[40] Mace T.A.R., Syed A., Bhandari S. (2013). Iron (III) isomaltoside 1000. Expert Rev. Hematol..

[41] Bhandari S., Kalra P.A., Kothari J., Ambühl P.M., Christensen J.H., Essaian A.M., Thomsen L.L., MacDougall I.C., Coyne D.W. (2015). A randomized, open-label trial of iron isomaltoside 1000 (Monofer^®^) compared with iron sucrose (Venofer^®^) as maintenance therapy in haemodialysis patients. Nephrol. Dial. Transplant..

[42] Kalra P.A., Bhandari S., Agarwal D., Wirtz G., Klauser-Braun R., Thomsen L.L., Coyne D.W. (2016). A randomized trial of iron isomaltoside 1000 versus oral iron in non-dialysis-dependent chronic kidney disease patients with anaemia. Nephrol. Dial. Transplant..

[43] Ottenjann M., Weingart C., Arndt G., Kohn B. (2006). Characterization of the Anemia of Inflammatory Disease in Cats with Abscesses, Pyothorax, or Fat Necrosis. J. Veter- Intern. Med..

[44] Wessling-Resnick M. (2010). Iron homeostasis and the inflammatory response. Annu. Rev. Nutr..

[45] Alfrey A.C., Hammond W.S. (1990). Renal iron handling in the nephrotic syndrome. Kidney Int..

[46] Nemeth E., Rivera S., Gabayan V., Keller C., Taudorf S., Pedersen B.K., Ganz T. (2004). IL-6 mediates hypoferremia of inflammation by inducing the synthesis of the iron regulatory hormone hepcidin. J. Clin. Investig..

[47] Wrighting D.M., Andrews N.C. (2006). Interleukin-6 induces hepcidin expression through STAT3. Blood.

[48] Pietrangelo A., Dierssen U., Valli L., Garuti C., Rump A., Corradini E., Ernst M., Klein C., Trautwein C. (2007). STAT3 Is Required for IL-6-gp130–Dependent Activation of Hepcidin In Vivo. Gastroenterology.

[49] Falzacappa M.V., Vujic S.M., Kessler R., Stolte J., Hentze M.W., Muckenthaler M.U. (2007). STAT3 mediates hepatic hepcidin expression and its inflammatory stimulation. Blood.

[50] Naigamwalla D.Z., Webb J.A., Giger U. (2012). Iron deficiency anemia. Can. Vet. J..

[51] Silverberg D.S., Wexler D., Blum M., Wollman Y., Sheps D., Iaina A., Schwartz D., Keren G. (2004). The Interaction between Heart Failure, Renal Failure and Anemia – The Cardio-Renal Anemia Syndrome. Blood Purif..

[52] Schwenk M.H. (2010). Ferumoxytol: A New Intravenous Iron Preparation for the Treatment of Iron Deficiency Anemia in Patients with Chronic Kidney Disease. Pharmacother. J. Hum. Pharmacol. Drug Ther..

[53] Vadhan-Raj S., Strauss W., Ford D., Bernard K., Boccia R., Li J. (2014). Allen LFEfficacy and safety of IV ferumoxytol for adults with iron deficiency anemia previously unresponsive to or unable to tolerate oral iron. Am. J. Hematol..

[54] Ganz T., Nemeth E. (2012). Hepcidin and iron homeostasis. Biochim. Biophys. Acta.

[55] Ghoti H., Rachmilewitz E.A., Simon-Lopez R., Gaber R., Katzir Z., Konen E., Kushnir T., Girelli D., Campostrini N., Fibach E. (2012). Evidence for tissue iron overload in long-term hemodialysis patients and the impact of withdrawing parenteral iron. Eur. J. Haematol..

[56] Singh A., Patel T., Hertel J., Bernardo M., Kausz A., Brenner L. (2008). Safety of Ferumoxytol in Patients with Anaemia and CKD. Am. J. Kidney Dis..

[57] Romeu M., Nogues R., Marcas L., Sánchez-Martos V., Mulero M., Martinez-Vea A., Mallol J., Giralt M. (2010). Evaluation of oxidative stress biomarkers in patients with chronic renal failure: A case control study. BMC Res. Notes.

[58] Caimi G., Carollo C., Hopps E., Montana M., Presti R.L. (2013). Protein oxidation in chronic kidney disease. Clin. Hemorheol. Microcirc..

[59] Floccari F., Aloisi C., Crasci E., Sofi T., Campo S., Tripodo D., Criseo M., Frisina N., Buemi M. (2005). Oxidative stress in uremia. Med. Res. Rev..

[60] Witko-Sarsat V., Friedlander M., Khoa T.N., Capeillère-Blandin C., Nguyen A.T., Canteloup S., Dayer J.M., Jungers P., Drüeke T., Descamps-Latscha B. (1998). Advanced oxidation protein products as novel mediators of inflammation and monocyte activation in chronic renal failure. J. Immunol..

[61] Kao M.P.C., Ang D.S.C., Pall A., Struthers A.D. (2010). Oxidative stress in renal dysfunction: Mechanisms, clinical sequelae and therapeutic options. J. Hum. Hypertens.

[62] Ganguli A., Kohli H.S., Khullar M., Gupta K.L., Jha V., Sakhuja V. (2009). Lipid Peroxidation Products Formation with Various Intravenous Iron Preparations in Chronic Kidney Disease. Ren. Fail..

[63] Sağlam F., Cavdar C., Uysal S., Cavdar Z., Camsari T. (2007). Effect of Intravenous Iron Sucrose on Oxidative Stress in Peritoneal Dialysis Patients. Ren. Fail..

[64] Zager R.A., Johnson A.C., Hanson S.Y., Wasse H. (2002). Parenteral iron formulations. A comparative toxicologic analysis and mechanisms of cell injury. Am. J. Kidney Dis..

[65] Bailie G.R., Schuler C., Leggett R.E., Li H.-D., Patadia H., Levin R. (2013). Oxidative effect of several intravenous iron complexes in the rat. BioMetals.

[66] Agarwal R., Vasavada N., Sachs N.G., Chase S. (2004). Oxidative stress and renal injury with intravenous iron in patients with chronic kidney disease. Kidney Int..

[67] Kuo K.-L., Hung S.-C., Lee T.-S., Tarng D.-C. (2014). Iron Sucrose Accelerates Early Atherogenesis by Increasing Superoxide Production and Upregulating Adhesion Molecules in CKD. J. Am. Soc. Nephrol..

[68] Boudina S., Sena S., Theobald H., Sheng X., Wright J.J., Hu X.X., Aziz S., Johnson J.I., Bugger H., Zaha V.G. (2007). Mitochondrial Energetics in the Heart in Obesity-Related Diabetes: Direct Evidence for Increased Uncoupled Respiration and Activation of Uncoupling Proteins. Diabetes.

[69] Fink B.D., Herlein J.A., Almind K., Cinti S., Kahn C.R., Sivitz W.I. (2007). Mitochondrial proton leak in obesity-resistant and obesity-prone mice. Am. J. Physiol. Integr. Comp. Physiol..

[70] Bhargava P., Schnellmann R.G. (2017). Mitochondrial energetics in the kidney. Nat. Rev. Nephrol..

[71] Che R., Yuan Y., Huang S., Zhang A. (2014). Mitochondrial dysfunction in the pathophysiology of renal diseases. Am. J. Physiol. Physiol..

[72] Liu S., Soong Y., Seshan S.V., Szeto H.H. (2014). Novel cardiolipin therapeutic protects endothelial mitochondria during renal ischemia and mitigates microvascular rarefaction, inflammation, and fibrosis. Am. J. Physiol. Physiol..

[73] Lan R., Geng H., Singha P.K., Saikumar P., Böttinger E.P., Weinberg J.M., Venkatachalam M.A. (2016). Mitochondrial Pathology and Glycolytic Shift during Proximal Tubule Atrophy after Ischemic AKI. J. Am. Soc. Nephrol..

[74] Ren X., Zou L., Zhang X., Branco V., Wang J., Carvalho C., Holmgren A., Lu J. (2017). Redox Signaling Mediated by Thioredoxin and Glutathione Systems in the Central Nervous System. Antioxid. Redox Signal..

[75] Pope S., Land J.M., Heales S.J. (2008). Oxidative stress and mitochondrial dysfunction in neurodegeneration: Cardiolipin a critical target?. Biochim. Biophys. Acta.

[76] Petrosillo G., Moro N., Ruggiero F.M., Paradies G. (2009). Melatonin inhibits cardiolipin peroxidation in mitochondria and prevents the mitochondrial permeability transition and cytochrome c release. Free Radic. Boil. Med..

[77] Brooks C., Wei Q., Cho S.G., Dong Z.I. (2009). Regulation of mitochondrial dynamics in acute kidney injury in cell culture and rodent models. J. Clin. Investig..

[78] Zhan M., Usman I.M., Sun L., Kanwar Y.S. (2015). Disruption of renal tubular mitochondrial quality control by Myo-inositol oxygenase in diabetic kidney disease. J. Am. Soc. Nephrol..

[79] Bayir H., Fadeel B., Palladino M.J., Witasp E., Kurnikov I.V., Tyurina Y.Y., Tyurin V.A., Amoscato A.A., Jiang J., Kochanek P.M. (2006). Apoptotic interactions of cytochrome c: Redox flirting with anionic phospholipids within and outside of mitochondria. Biochim. Biophys. Acta.

